# A Network Pharmacology Approach to Predict the Proangiogenesis Mechanism of Huangqi-Honghua Herb Pair after Cerebral Ischemia

**DOI:** 10.1155/2021/9834856

**Published:** 2021-04-14

**Authors:** Jinyi Cao, Lu Lei, Kai Wang, Jing Sun, Yi Qiao, Jialin Duan, Chao Zhao, Jia Cui, Zhijun Feng, Jing-wen Wang, Aidong Wen, Zhifu Yang

**Affiliations:** ^1^Department of Pharmacy, Xijing Hospital, Fourth Military Medical University, Xi'an 710032, China; ^2^School of Basic Medicine and Clinical Pharmacy, China Pharmaceutical University, Nanjing 211198, China; ^3^College of Pharmacy, Shaanxi University of Chinese Medicine, Xianyan 712046, China; ^4^Department of Anesthesiology, Northwest Women's and Children's Hospital, Xi'an 710061, China

## Abstract

**Objective:**

Huangqi-Honghua herb pair is known for its medicinal value to treat Qi deficiency and blood stasis syndrome with a long history in clinical practice. To understand its possible mechanism in a systematic study, a network pharmacological method was addressed.

**Methods:**

Detailed information on the HH compounds was obtained from two public databases, and oral bioavailability (OB) and drug-like (DL) of the compounds were evaluated. A correlation between HH compounds, its potential targets, and known targets was extrapolated, and the herb-compound-target-disease (H-C-T-D) network was established. Next, the pathway enrichment and essential genes were analyzed. Then, three key genes (VEGFA, VEGFR2, and eNOS), highly associated with angiogenesis, were screened and verified through western blot assay.

**Results:**

Out of 276 compounds, 21 HH compounds and 78 target genes regulating the major pathways associated with CI in the network are analyzed. The bioactive compounds in HH were active in various signal transduction pathways such as the toll-like receptor signaling pathway, VEGF signaling pathway, TNF signaling pathway, and HIF-1 signaling pathway are important pathways that may regulate anti-inflammatory, antiapoptotic, immune correlation, and antioxidative effects. The core genes are PTGS2, TNF, NOS2, IL6, BCL2, IL1B, SOD2, NOS3, SOD1, MMP9, and VEGFA. The in vitro results suggested that HH treatment could significantly elevate the expression of proangiogenic genes such as VEGFA, VEGFR2, and eNOS compared with OGD groups.

**Conclusions:**

Our results predict that HH may regulate the expression of VEGFA, VEGFR2, and eNOS via the VEGF and HIF-1 signaling pathway to promote angiogenesis and alleviate cerebral ischemia injury.

## 1. Introduction

Ischemic stroke (cerebral ischemia, CI) has become a major public health concern with morbidity, mortality, and health care costs [1]. Irrespective of the current standard of care options (e.g., mechanical or pharmacological (tissue plasminogen activator, tPA) reperfusion) available in the clinical practice, there is an unmet need for ischemic stroke because of the narrow treatment time window [2]. Thus, there is an unmet need for developing regenerative medicine to address CI and its secondary effects. One of the devastating consequences of CI is decreased blood supply to the injured brain, and it leads to the functional impairment of the brain tissue in the affected area. Developing an effective strategy to increase the blood supply to the ischemic area may potentially rescue the brain tissue from death and may improve the recovery rate of the patients [3]. Previous studies have indicated that the higher the vascular density of ischemic penumbra, the longer the survival time of ischemic stroke patients [4, 5]. Therefore, proangiogenesis may be a valid antistroke strategy to address current clinical hurdles associated with a stroke patient, which has the potential to repair and rewire the injured nerve connections.

Traditional Chinese medicine (TCM) has been shown to be beneficial in promoting developmental angiogenesis, which highlights “reinforcing qi to enrich the blood, dispelling stasis to promote regeneration” theory by associating with angiogenesis together. Radix Astragali (Huangqi) is the dry root of *Astragalus membranaceus* (Fisch.) Bge, which has been commonly used in patients with stroke or chronic weakness in China because it can enhance Qi and dispel blood stasis, with antioxidation, anti-inflammatory, immunomodulation, and anticancer effects in modern medical research [6, 7]. *Carthamus tinctorius* L. (Honghua), a dried flower, often used to promote blood circulation, dispel blood stasis, and relieve pain [8, 9]. Huangqi-Honghua (HH) herb pair has been used widely in clinical practice for treating Qi deficiency and blood stasis syndrome, especially cerebrovascular and cardiovascular diseases, with a long history, such as Buyang Huanwu decoction, a well-known TCM formula [10–12]. In our previous study, by using a rat model of middle cerebral artery occlusion (MCAO), we have validated the synergetic effect of hydroxysafflor yellow A and astragaloside IV (main constituents of HH) in removing blood stasis (QDBS) syndrome [13].

Network pharmacology follows the dogma of “drug-targets-gene-disease,” to predict drug targets and improve the efficiency of drug discovery effort. Network pharmacology maintains integrity and systematic characteristic, similar to the principle of traditional Chinese medicine that involves multiple components and multiple targets [14]. Network pharmacology has become a novel and efficient way to interpret the complex pharmacological mechanism of TCMs scientifically, particularly, formulae orchestration [15].

**Figure 1:**
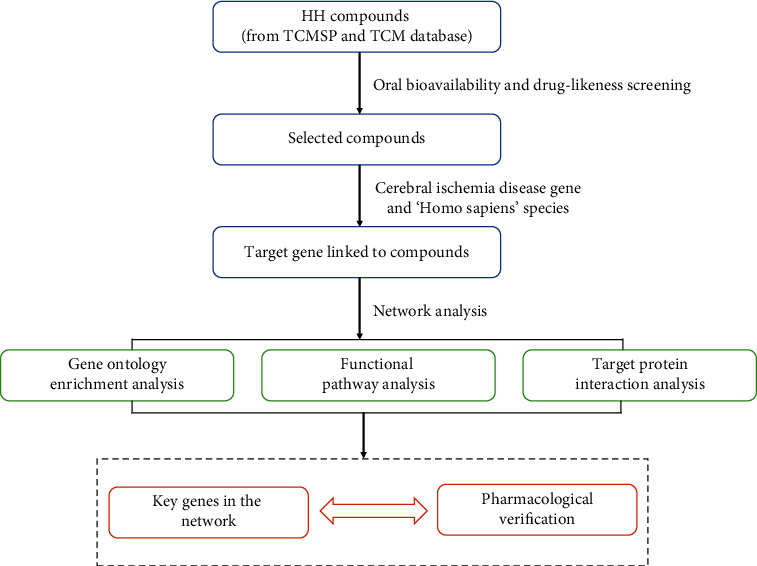
Workflow of network pharmacology analysis.

Despite a few studies that have proved the protective effect of HH in the cerebral hemisphere, pharmacology-based prediction of complete profiling of bioactive components and the network of their target pathways has not fully been elucidated. Thus, in the present study, we have explored the proangiogenesis mechanisms of HH in the etiology of CI. In this study, we have established the herb-compound-target-disease (H-C-T-D) networks by utilizing the network pharmacology approach. Furthermore, to demonstrate angiogenesis, one key pathway of HH in cerebral protection, we have identified three key targets, which is highly associated with angiogenesis, through immunoblot assay. The workflow is shown in Figure 1.

## 2. Materials and Methods

### 2.1. Chemical Databases

Information on the herbs and compounds related to HH were collected from two botanical chemistry databases: TCM Database of @Taiwan (http://tcm.cmu.edu.tw/) and the traditional Chinese Medicine system Pharmacology Database (TCMSP, http://ibts.hkbu.edu.hk/LSP/tcmsp.php). The chemical structures, 2D structure, molecular weight, and chemical number are reported on ChEMBL (http://www.ebi.ac.uk/chembl/) and NCBI PubChem (http://puchem.nvbi.nlm.nih.gov/).

### 2.2. Oral Bioavailability and Drug-Likeness of HH Components

All compounds in HH were evaluated for their oral bioavailability (OB) and drug-likeness (DL). OB is one of the most common pharmacokinetic parameters in the drug screening process, which explains the percentage of an oral drug that remains unchanged after entering into the systemic circulation. In addition, OB provides a clue for the fusion of the ADME process. DL measures the extent of the “drug-like” property of a compound and determines whether the compound influences absorption, distribution, metabolism, and excretion (ADME) in the human body like an approved drug. In this study, chemicals only with an OB ≥30 and DL ≥0.18 (recommended by TCMSP database) were considered for further valuation [16].

### 2.3. Collection Target Genes Relevant to the Screened Compound

UniProt (http://www.uniprot.org/) platform, by setting the filter to “*Homo sapiens*,” was used to identify the target genes, closely relevant to chemicals. Meanwhile, information on target genes related to the CI was obtained from the therapeutic target database (TTD; http://www.bidd.Nus.edu.sg/group/cjttd/TTD%20HOME.asp) and DisGeNETv6.0 (http://www.disgenet.org/web/DisGeNET/menu/home). Finally, we assigned the gene names and ID by constructing herbs-ingredients-targets relationship dataset for HH.

### 2.4. Network Construction and Pathway Analyses

The herb-compound-target-disease (H-C-T-D) networks were established using Cytoscape 3.3.0, JAVA software. Functional pathways annotation related to CI and enrichment evaluation was evaluated using the Kyoto Encyclopedia of Genes (KEGG), Genomes (GO), and the Database for Annotation, Visualization, and Integrated Discovery (DAVID) version 6.8 (http://david.ncifcrf.gov/).

### 2.5. Protein-Protein Interaction (PPI) Data

PPI data were collected from the Search Tool for the Retrieval of Interacting Genes (STRING) database (http://string-db.org/, 11.0 version), and possible protein-protein interactions were predicted. We chose and showed the top 20 target genes with confidence score >0.9 as the core genes for CI.

### 2.6. Pharmacological Experiment

#### 2.6.1. Materials

HH granules (Huangqi :  Honghua = 5 : 1, previous research has confirmed) were obtained from Guangdong Yifang Pharmaceuticals Co., Ltd. (product batch: 20170606). The rat brain microvascular endothelial cells (BMECs) were purchased from the Cell Biologics Company (#C57-6023, Chicago, IL). Primary antibodies against *β*-actin, VEGFA, eNOS, MMP9, IL6, and secondary antibody were all purchased from Abcam Technology (Cambridge, UK). All other reagents are analytical reagents and commercially available.

#### 2.6.2. Cell Culture and Treatments

Cells were divided into three groups: control group, OGD group, and OGD group treated with HH (100 *μ*g/mL). Oxygen-glucose deprivation (OGD) was established as follows: cells were rinsed once with glucose-free DMEM (Gibco, Rockville, MD) and transferred to an anaerobic chamber (Forma Scientific, Waltham, MA) containing a gas mixture composed of 7% CO_2_ and 93% N_2_ for 6 h at 37°C. Then, the cells were returned to the normal culture condition. Control BMECs were cultured in complete DMEM under normal conditions. HH groups were treated with HH (dissolved in complete DMEM and filtered with a 0.22 *μ*m membrane filter) for 12 h. The control group was treated without HH.

#### 2.6.3. Western Blot Assay

BMECs were lysed with cold RIPA buffer (Rockford, IL, USA) for 30 min. The whole cell lysates were separated by 10% sepharose gel, and PVDF membranes (Millipore, USA) were used to transfer protein. The membranes were incubated with 5% bovine serum albumin (BSA) and overnight with primary antibodies against VEGFA (1 : 1000, rabbit), VEGFR2 (1 : 1000, rabbit), and eNOS (1 : 1000, rabbit) at 4°C. After that, membranes were incubated with secondary antibody at a 1 : 5000 dilution at 37°C for 1 h. After ECL-Plus reagent (Santa Cruz, USA) treatment, the blots were analyzed with Quantity One System image analysis software (Bio-Rad, USA).

#### 2.6.4. Statistical Analysis

The results were expressed as mean ± standard deviation (S.D.) and analyzed with one-way analysis of variance (ANOVA), followed by a Tukey's post hoc test was used for analyzing differences between groups. *p* < 0.05 presented statistical significance.

## 3. Results

### 3.1. Identification of Active Compounds in HH

**Figure 2:**
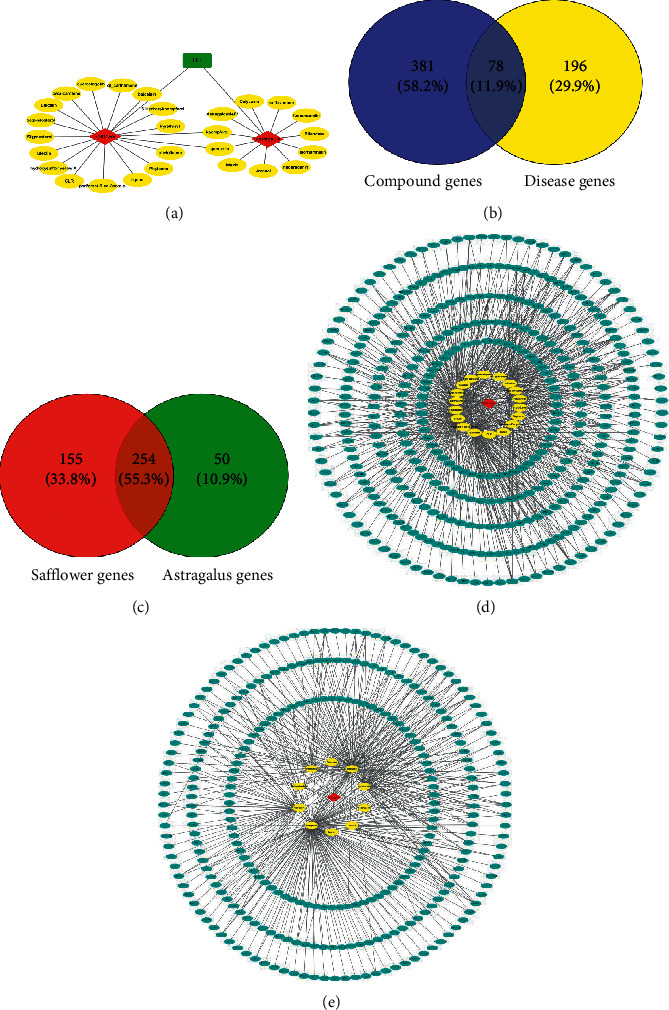
Linkage of target compounds and target genes. (a) The network of herbal medicine-compound in HH. (b) The Venn diagram of the compound genes and disease genes. (c) The Venn diagram of the target genes for safflower (Honghua) and astragalus (Huangqi). (d, e) The pharmacology networks of two herb medicines (red diamonds) which connect with target genes (blue ellipses) compounds (yellow ellipses).

HH consists of a total of 276 compounds, 87 in Astragalus (Huangqi) and 189 in Safflower (Honghua). All 276 compounds were screened with OB and DL, out of which, only 42 compounds were recommended for the final screening. The remaining compounds were discarded. The therapeutic effect of a few selected compounds on CI was confirmed through the related literature search. Therefore, preselected active ingredients were added manually, including hydroxysafflor yellow A and astragaloside IV [17–19]. After that, the repeated ingredients and the ingredients with the unclear target are deleted. The selected 27 compounds from HH is shown in Table 1 and Figure 2(a).

### 3.2. Compounds-Targets Analysis

The active ingredients of HH obtained from the screening assay were predicted using TCMSP and CTD database (https://ctdbase.org/). The repeated targets were deleted, and the unclear targets were eliminated. A total of 459 predicted target genes were obtained related to the 27 identified compounds in HH (Figures 2(a) and 2(c)). The compound-target gene network includes 683 nodes and 1146 edges as shown in Figure 2(e). The nodes (the points of communication or redistribution) and edges (the lines of communication joining the nodes) of HH are listed in Table 2. Kaempferol is the compound of maximum interactions with target genes.

### 3.3. Network Construction and Analysis

**Figure 3:**
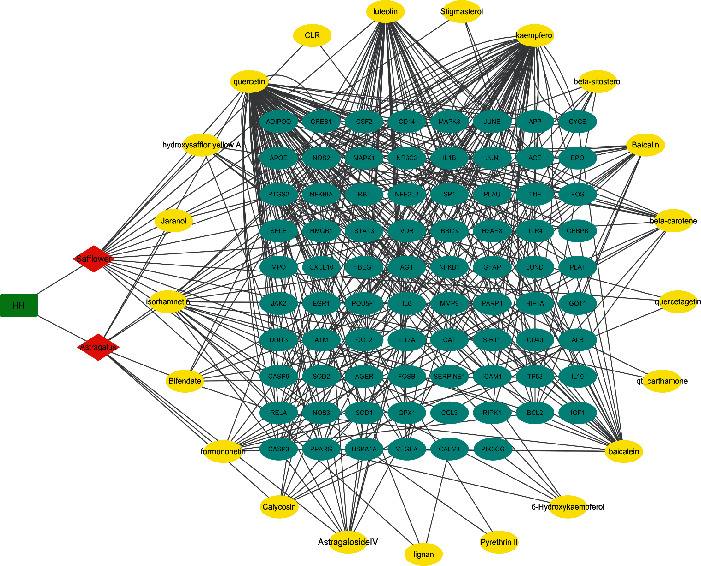
The herb-compound-gene network for HH.

\Out of the 459 matching HH-associated genes and 274 CI-related genes, we streamlined 78 overlapping genes while constructing the gene network (Figure 2(b), Table 3). This gene network included 101 nodes (2 Chinese herb medicines, 21 compounds, and 78 target genes) and 367 edges (Figure 3), and 21 compounds were categorized as flavonoids, lignans, terpene, sterols, triterpenoids, and their glycosides. Particularly, 13 compounds (astragaloside IV, calycosin, formononetin, bifendate, isorhamnetin, hydroxysafflor yellow A, quercetin, luteolin, kaempferol, *β*-sitosterol, baicalin, *β*-carotene, and pyrethrin II) were related to more than five genes, and 18 genes (PTGS2, TNF, CASP3, NOS2, RELA, PPARG, IL6, BCL2, NFKBIA, IL1B, CAT, MAPK1, CASP9, MAPK3, TP53, PARP1, SOD2, NOS3, NFE2L2, SOD1, MMP9, ICAM1, JUN, and VEGFA) related to more than five compounds. Therefore, we speculate that these compounds and genes might be key nodes in this network. The compound-target gene network will be conducive to interpretation and prediction the “multicompound, multitarget, and multipathway” of HH.

### 3.4. Pathway and GO Term Enrichment Analysis

**Figure 4:**
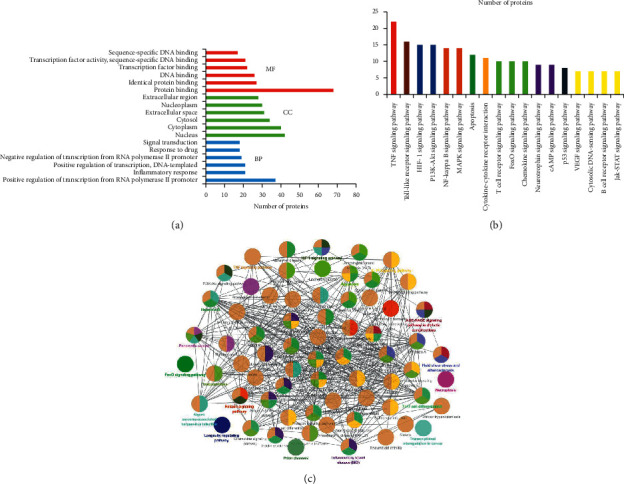
KEGG pathway and GO analysis by DAVID database. (a) GO analysis of candidate targets. Database showed the five remarkably enriched items in the biological processes (BP), cell component (CC), and molecular function (MF). (b) KEGG pathways of target genes. (c) Main functional annotation clusters by biocarta analysis.

To explore the signaling pathway and functions of the identified target genes, 78 candidate targets were analyzed by GO and enriched by the KEGG pathway. The top 6 enriched conditions were found to be involved in the biological process (BP), cell component (CC), and molecular function (MF) (Figure 4(a)). According to the results of GO enrichment analysis, the positive regulation of transcription from RNA polymerase II promoter, inflammatory response, response to lipopolysaccharide, aging, response to drug, signal transduction etc., dominates the biological process. Forty-two targets, the largest proportion, account for cell components. Screening of *p* < 0.05 signal pathway by KEGG enrichment analysis eliminates signaling pathways that are not associated with CI and get the top 18 signaling pathways (Figure 4(b) and Table 4). Therefore, we speculate that the TNF signaling pathway, toll-like receptor signaling pathway, HIF-1 signaling pathway, PI3K-Akt signaling pathway, NF-kappa B signaling pathway, and VEGF signaling pathway may be the important pathways to exert their synergistic effects against cerebral ischemia injury.

### 3.5. PPI Networks

**Figure 5:**
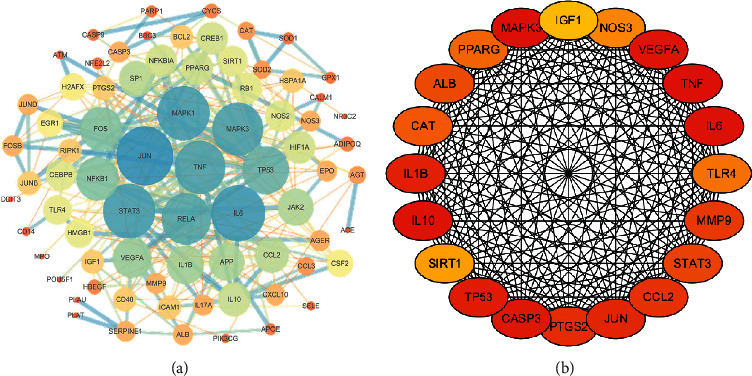
Protein-protein interaction (PPI) networks of active ingredients of HH for the treatment of cerebral ischemia. (a) Each node represents the relevant gene, the edge. Means line thickness indicates the strength of data support. (b) Center top 20 genes in the PPI network, the darker the color, the higher the score.

In the PPI relationships network, we found 78 nodes and 1429 edges (Figure 5(a)). The top 20 core genes, including tumor necrosis factor (TNF), interleukin 6 (IL6), MAP kinase-activated protein kinase 3 (MAPK3), vascular endothelial growth factor A (VEGFA), interleukin 10 (IL10), matrix metalloproteinase-9 (MMP9), and endothelial nitric oxide synthase (eNOS/NOS3) were selected (Figure 5(b)). Darker color scheme denotes a higher score.

### 3.6. Experimental Validation

**Figure 6:**
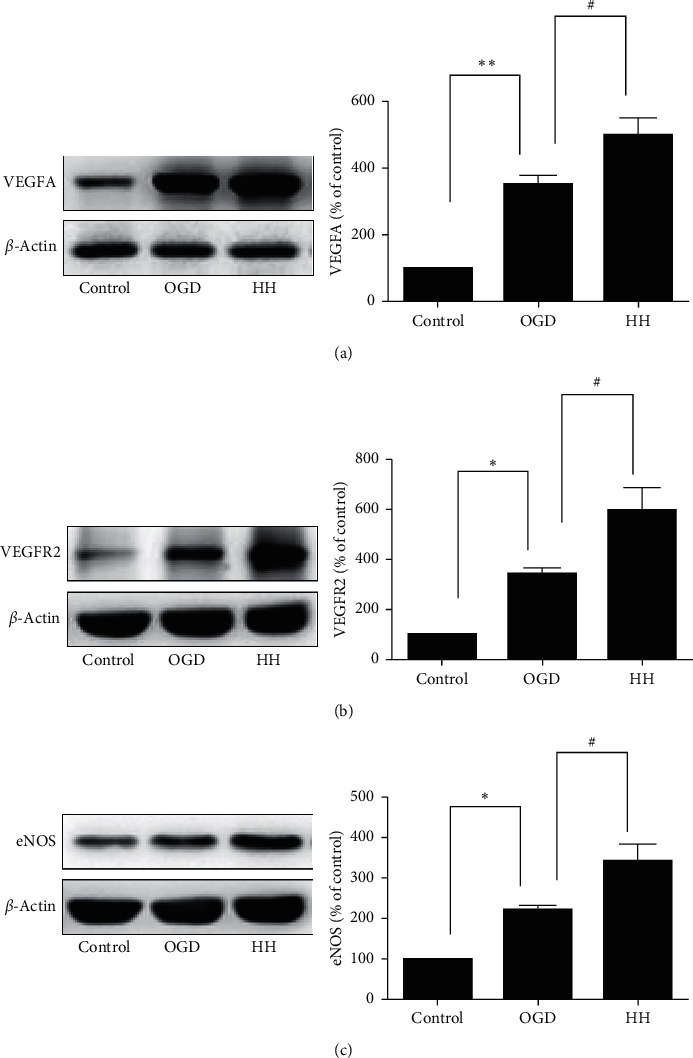
Effects of HH on the levels of VEGFA, VEGFR2, and eNOS in BMECs. (*∗p* < 0.05 and *∗∗p* < 0.01 compared with the control group; #*p* < 0.05 compared with the OGD group).

To confirm the results from the network and to verify that angiogenesis is indeed one of the key pathways of HH in cerebral protection, we selected three targets related to angiogenesis (VEGFA, VEGFR2, and eNOS) for pharmacological validation (Figure 6). Western blot analysis indicated that treatment with HH could significantly increase the expression of VEGFA, VEGFR2, and eNOS, compared with the OGD group (*∗p* < 0.05, *∗∗p* < 0.01 compared with control group, and #*p* < 0.05 compared with OGD group).

## 4. Discussion

Multicompound TCMs, with multiple biological targets, are the prime focus in Chinese clinical practice for more than thousand years. Surprisingly, TCMs are known to target multiple biological pathways to defend against diseases. However, the traditional method of usage of TCMs barely provides insights into the biological complexity of compounds, its biological targets, and associated disease, which limits the development and reformulation of TCMs. Lately, network pharmacology approach of analyzing TCMs gains more popularity that dissects the pharmacological mechanism of action [20–22].

In the current study, a network pharmacology analysis of HH identified 2 herbs, 21 compounds, as well as 78 target gene-regulated major pathways associated with CI. Through the pathway enrichment analysis, we found that the targets of active ingredients in HH against cerebral ischemia injury mainly participate in numerous signal transduction pathways such as TNF signaling pathway, toll-like receptor signaling pathway, HIF-1 signaling pathway, PI3K-Akt signaling pathway, and VEGF signaling pathway. These key pathways may regulate anti-inflammatory, antiapoptotic, immune correlation, and antioxidative effects. Furthermore, the PPI system analysis indicates these genes played vital roles in CI (Figure 5(b)), such as TNF, IL6, MAPK3, VEGFA, IL10, and MMP9. In addition, three key targets (VEGFA, VEGFR2, and eNOS), predicted in the network, highly related to angiogenesis were verified by using western blot assay.

According to the TCM theory, Qi and blood dysfunction is one key pathogenesis of CI. In the clinic, Qi-tonifying drugs, blood-activating drugs, and reinforcing Qi and activating blood drugs team were usually treated for CI with significant efficacy. A fewer side effects [23, 24] of these drugs could promote postischemic angiogenesis to recovery the cerebral blood flow and tissue perfusion, supporting the survival of neurons and neural progenitor cells and promoting long-term functional recovery [25, 26]. Huangqi-Honghua (HH), a common reinforcing Qi and activating blood drugs team, has been used widely in clinical practice for treating Qi deficiency and blood stasis syndrome, especially, CI with Qi deficiency and blood stasis [7, 27]. Based on the available data, there are 276 compounds found in HH and categorized into flavonoids, lignans, terpene, sterols, triterpenoids, and their glycosides. After screening with OB and DL, we selected 21 compounds that were correlated with 78 human protein targets for CI. The top 10 key compounds related to more than five genes were found to be astragaloside IV, calycosin, formononetin, bifendate, isorhamnetin, hydroxysafflor yellow A, quercetin, luteolin, kaempferol, and beta-sitosterol. Uniformly, hydroxysafflor yellow A and astragaloside IV are in the list. Our previous study has indicated that HH could significantly ameliorate cerebral ischemia injury, and the mainly effective compounds are hydroxysafflor yellow A and astragaloside IV [13]. Moreover, in a recent study, astragaloside IV also has also been shown to possess anti-inflammatory and antioxidant activation potential [28]. Through the network analysis results, kaempferol has the maximum interactions with target genes, especially can attenuate neuroinflammation and blood brain barrier dysfunction to improve neurological deficits in cerebral I/R rats by regulating the NK-*κ*B pathway [29]. However, further studies need to understand if kaempferol is the most important pharmacodynamics substance. Calycosin, which strikingly downregulates HUVEC TGF-betal, ICAM1, and RAGE expression [30], has antitumor, neuroprotective, anti-inflammatory, and proangiogenesis effects [31]. *In vivo* study also revealed quercetin to have anti-inflammatory effects by inhibiting oxidative stress and cytokine production [32]. Formononetin exert its therapeutic effects by regulating these biological processes such as hormone metabolism, apoptosis, cell communication, and signal transduction [33]. These findings were consistent with the results from the active component target and indicate that the main components of HH are effective for treating target disease(s).

Angiogenesis is similar to “reinforcing qi to enrich blood, Dispelling stasis to promote regeneration” theory and regulated by angiogenesis inducers and inhibitors. Angiogenesis involves basilar membrane degradation, chemotactic migration, and proliferation of EC (endothelial cell) and EPC (endothelial progenitor cell). In the PPI system analysis, top 20 center genes were recognized, and among these genes, VEGFA and eNOS are highly correlated with angiogenesis. Over the few decades, vascular endothelial growth factors (VEGFs) and their receptors (VEGFRs) have been regarded as the principal drivers of angiogenesis and the development and maintenance of vascular system [34]. VEGFA (commonly known as VEGF) is the prototype member of the VEGF family of proteins, which stimulate angiogenesis in blood vascular endothelial cells via receptor-binding with VEGFR2 [35, 36]. Previous report suggests that overexpressed IncRNA ANRIL upregulates VEGF and promotes angiogenesis by activating NF-*κ*B pathway in CI rats [37]. Endothelial nitric oxide synthase (eNOS/NOS3) provides continuous local production of nitric oxide (NO), a crucial angiogenesis mediator and effector [38]. The increase in NO production via the upregulation of NOS3 by VEGF indicates that the angiogenic effect of VEGF seems to be mediated by NO [39]. Therefore, we verified the function of three essential genes (VEGFA, VEGFR2, and eNOS) using western blot assay, and the results show that VEGFA, VEGFR2, and eNOS were upregulated in BMECs with oxygen-glucose deprivation treatment with HH, which is beneficial for angiogenesis.

According to the KEGG pathway analysis, these three target genes interact mainly with HIF-1 signaling pathway and VEGF signaling pathway. Low oxygenation concentrations in tissues (hypoxia) often trigger angiogenesis [40], which can be initiated independently of VEGF-related pathways, as well as lead to expression of multiple growth factors such as VEGF and NOS, via the HIF pathway [41]. HIF-regulated proangiogenic factors can increase vascular permeability, endothelial cell proliferation, migration, adhesion, and tube formation [42]. Therefore, we speculate that HH can regulate VEGFA, VEGFR2, and eNOS via the pathways associated with promote angiogenesis and alleviate cerebral ischemia injury. However, our current study still have some limitations, the compounds in the database may be incomplete, and it is incomprehensive that the vitro experiments only be used to confirm the results from the network. In the future, further studies require to design and implement a range of pharmacological experiment to investigate the possible pathway that controls proangiogenesis.

In summary, network pharmacology analysis of HH identified 2 herbs, 21 compounds, and 78 target gene-regulated major pathways associated with CI. The bioactive compounds in HH mainly participate in numerous signal transduction pathways such as TNF signaling pathway, toll-like receptor signaling pathway, HIF-1 signaling pathway, PI3K-Akt signaling pathway, and VEGF signaling pathway, and these important pathways may regulate anti-inflammatory, antiapoptotic, immune correlation, and antioxidative effects. Furthermore, through the pharmacological experiment, we predict that HH can regulate VEGFA, VEGFR2, and eNOS via the HIF-1 signaling pathway and VEGF signaling pathway to promote angiogenesis and alleviate cerebral ischemia injury.

## Figures and Tables

**Table 1:** A list of the final selected compounds among the two herbal medicines for network analysis.

No.	Compound	OB (%)	DL	Herb
1	Kaempferol	41.88	0.24	Safflower, astragalus
2	Quercetin	46.43	0.28	Safflower, astragalus
3	Lignan	43.32	0.65	Safflower
4	Phytoene	39.56	0.5	Safflower
5	Phytofluene	43.18	0.5	Safflower
6	Pyrethrin II	48.36	0.35	Safflower
7	6-Hydroxykaempferol	62.13	0.27	Safflower
8	Baicalein	33.52	0.21	Safflower
9	Qt_carthamone	51.03	0.2	Safflower
10	Quercetagetin	45.01	0.31	Safflower
11	Beta-carotene	37.18	0.58	Safflower
12	Baicalin	40.12	0.75	Safflower
13	Beta-sitosterol	36.91	0.75	Safflower
14	Poriferast-5-en-3beta-ol	36.91	0.75	Safflower
15	Stigmasterol	43.83	0.76	Safflower
16	Luteolin	36.16	0.25	Safflower
17	CLR	37.87	0.68	Safflower
18	Hydroxysafflor yellow A	4.77	0.68	Safflower
19	Mairin	55.38	0.78	Astragalus
20	Jaranol	50.83	0.29	Astragalus
21	Hederagenin	36.91	0.75	Astragalus
22	Isorhamnetin	49.6	0.31	Astragalus
23	Bifendate	31.1	0.67	Astragalus
24	Formononetin	69.67	0.21	Astragalus
25	Isoflavanone	109.99	0.3	Astragalus
26	Calycosin	47.75	0.24	Astragalus
27	Astragaloside IV	17.74	0.15	Astragalus

**Table 2:** Nodes and edges of HH.

	Safflower	Astragalus
Compounds	18	11
Targets of compounds	409	304
Nodes	391	292
Edges	680	466
Number of maximum interactions	159	159
Compounds of maximum interactions with target genes	Kaempferol	Kaempferol

**Table 3:** A list of the compound and disease common target protein.

No.	Gene	Compounds
1	PTGS2	Lignan, pyrethrin II, 6-hydroxykaempferol, baicalein, qt_carthamone, quercetagetin, baicalin, kaempferol, luteolin, quercetin, jaranol, isorhamnetin, bifendate
2	TNF	Baicalein, beta-carotene, baicalin, kaempferol, luteolin, quercetin, hydroxysafflor yellow A, isorhamnetin, bifendate, Astragaloside IV, stigmasterol
3	CASP3	Baicalein, beta-carotene, baicalin, beta-sitosterol, kaempferol, luteolin, quercetin, isorhamnetin, formononetin, astragaloside IV
4	NOS2	6-Hydroxykaempferol, baicalein, baicalin, kaempferol, luteolin, quercetin, jaranol, isorhamnetin, bifendate
5	RELA	Safflower, baicalein, baicalin, kaempferol, luteolin, quercetin, isorhamnetin, bifendate, astragaloside IV
6	PPARG	6-Hydroxykaempferol, baicalein, quercetagetin, baicalin, kaempferol, quercetin, hydroxysafflor yellow A, isorhamnetin, formononetin
7	IL6	Baicalin, kaempferol, luteolin, quercetin, hydroxysafflor yellow A, isorhamnetin, bifendate, astragaloside IV
8	BCL2	Baicalein, beta-carotene, beta-sitosterol, kaempferol, luteolin, quercetin, isorhamnetin, formononetin
9	NFKBIA	Baicalein, baicalin, kaempferol, luteolin, quercetin, isorhamnetin, formononetin, astragaloside IV
10	IL1B	Baicalein, beta-carotene, baicalin, kaempferol, luteolin, quercetin, isorhamnetin, astragaloside IV
11	CAT	Beta-carotene, beta-sitosterol, kaempferol, luteolin, quercetin, isorhamnetin, bifendate
12	MAPK1	Calycosin, beta-carotene, baicalein, luteolin, kaempferol, quercetin
13	CASP9	Beta-sitosterol, kaempferol, luteolin, quercetin, isorhamnetin, formononetin
14	MAPK3	Baicalein, beta-carotene, kaempferol, luteolin, quercetin, calycosin
15	TP53	Baicalin, luteolin, quercetin, formononetin, kaempferol, beta-carotene
16	PARP1	Beta-sitosterol, kaempferol, luteolin, quercetin, isorhamnetin
17	JUN	Baicalein, beta-carotene, kaempferol, luteolin, quercetin
18	VEGFA	Baicalein, kaempferol, luteolin, quercetin, calycosin
19	NFE2L2	Kaempferol, luteolin, quercetin, astragaloside IV
20	SOD1	Kaempferol, luteolin, quercetin, astragaloside IV
21	MMP9	Baicalein, luteolin, quercetin, hydroxysafflor yellow A
22	ICAM1	Kaempferol, luteolin, quercetin, hydroxysafflor yellow A
23	SOD2	Beta-carotene, kaempferol, quercetin, astragaloside IV
24	NOS3	Luteolin, isorhamnetin, formononetin, quercetin
25	CSF2	Baicalein, kaempferol, luteolin, quercetin
26	CYCS	Baicalein, beta-sitosterol, quercetin, kaempferol
27	MPO	Baicalein, luteolin, quercetin, astragaloside IV
28	HIF1A	Kaempferol, luteolin, quercetin, formononetin
29	APP	Baicalein, kaempferol, quercetin
30	IL10	Stigmasterol, luteolin, bifendate
31	CCL2	Kaempferol, luteolin, quercetin
32	FOS	Kaempferol, luteolin, quercetin
33	NFKB1	Baicalein, luteolin, quercetin
34	STAT3	Luteolin, baicalein, quercetin
35	AGT	Isorhamnetin, quercetin
36	CD40	Luteolin, hydroxysafflor yellow A
37	EGR1	Luteolin, quercetin
38	CXCL10	Luteolin, quercetin
39	SELE	Kaempferol, luteolin
40	CEBPB	Baicalein, quercetin
41	TLR4	Baicalin, quercetin
42	EPO	Formononetin, calycosin
43	SERPINE1	Isorhamnetin, quercetin
44	JAK2	Quercetin
45	CCL3	Isorhamnetin
46	NR3C2	CLR
47	CALM1	Lignan
48	RB1	Baicalein
49	IGF1	Baicalein
50	APOE	Kaempferol
51	BBC3	Kaempferol
52	ATM	Kaempferol
53	CD14	Kaempferol
54	H2AFX	Kaempferol
55	RIPK1	Kaempferol
56	VDR	Kaempferol
57	PLAU	Baicalein
58	ADIPOQ	Beta-carotene
59	GOT1	Baicalin
60	PIK3CG	6-Hydroxykaempferol
61	HSPA1A	Quercetin
62	FOSB	Luteolin
63	GFAP	Luteolin
64	GPX1	Luteolin
65	HBEGF	Luteolin
66	IL17A	Luteolin
67	JUNB	Luteolin
68	JUND	Luteolin
69	DDIT3	Quercetin
70	SIRT1	Quercetin
71	HMGB1	Quercetin
72	ALB	Quercetin
73	PLAT	Quercetin
74	CREB1	Quercetin
75	ACE	Luteolin
76	AGER	Luteolin
77	SP1	Quercetin
78	POU5F1	Kaempferol

**Table 4:** Functions of potential target genes based on KEGG pathway analysis.

Pathway ID	Pathway classification	Term	Number of pathway genes	*p* value
hsa04668	Signal transduction	TNF signaling pathway	CCL2, CXCL10, CEBPB, FOS, JUN, JUNB, NFKBIA, RELA, CREB1, CASP3, CSF2, ICAM1, IL1B, IL6, MMP9, MAPK1, NFKB1, PIK3CG, PTGS2, RIPK1, SELE, TNF	1*E* − 21
hsa04620	Immune system	Toll-like receptor signaling pathway	CCL3, CXCL10, CD14, CD40, FOS, JUN, NFKBIA, RELA, IL1B, IL6, MAPK1, NFKB1, PIK3CG, RIPK1, TLR4, TNF	2.1*E* − 13
hsa04066	Signal transduction	HIF-1 signaling pathway	BCL2, RELA, EPO, HIF1A, IGF1, IL6, MAPK1, NOS2, NOS3, NFKB1, PIK3CG, SERPINE1, STAT3, TLR4, VEGFA	9.2*E* − 13
hsa04064	Signal transduction	NF-kappa B signaling pathway	ATM, BCL2, CD14, CD40, NFKBIA, RELA, ICAM1, IL1B, NFKB1, PLAU, PTGS2, RIPK1, TLR4, TNF	4.7*E* − 12
hsa04210	Cell growth and death	Apoptosis	ATM, BCL2, NFKBIA, RELA, CASP3, CASP9, CYCS, NFKB1, PIK3CG, RIPK1, TNF, TP53	3.4*E* − 11
hsa04660	Immune system	T-cell receptor signaling pathway	FOS, JUN, NFKBIA, RELA, CSF2, IL10, MAPK1, NFKB1, PIK3CG, TNF	0.0000010
hsa04010	Signal transduction	MAPK signaling pathway	CD14, DDIT3, FOS, JUN, JUND, RELA, CASP3, HSPA1A, IL1B MAPK1, MAPK3, NFKB1, TNF, TP53	0.0000023
hsa04115	Cell growth and death	p53 signaling pathway	ATM, BBC3, CASP3, CASP9, CYCS, IGF1, SERPINE1, TP53	0.0000065
hsa04068	Signal transduction	FoxO signaling pathway	ATM, CAT, IGF1, IL10, IL6, MAPK1, PIK3CG, STAT3, SIRT1, SOD2	0.0000120
hsa04151	Signal transduction	PI3K-Akt signaling pathway	BCL2, JAK2, RELA, CREB1, CASP9, EPO, IGF1, IL6, MAPK1, NOS3, NFKB1, PIK3CG, TLR4	0.0000140
hsa04722	Nervous system	Neurotrophin signaling pathway	BCL2, JUN, NFKBIA, RELA, CALM1, MAPK1, NFKB1, PIK3CG, TP53	0.0000390
hsa04370	Signal transduction	VEGF signaling pathway	CASP9, MAPK1, MAPK3, NOS3, PIK3CG, PTGS2, VEGFA	0.0000440
hsa04623	Immune system	Cytosolic DNA-sensing pathway	CXCL10, NFKBIA, RELA, IL1B, IL6, NFKB1, RIPK1	0.0000580
hsa04662	Immune system	B-cell receptor signaling pathway	FOS, JUN, NFKBIA, RELA, MAPK1, NFKB1, PIK3CG	0.0000890
hsa04062	Immune system	Chemokine signaling pathway	CCL2, CCL3, CXCL10, JAK2, NFKBIA, RELA, MAPK1, NFKB1, PIK3CG, STAT3	0.0001500
hsa04060	Signaling molecules and interaction	Cytokine-cytokine receptor interaction	CCL2, CCL3, CXCL10, CD40, CSF2, EPO, IL1B, IL10, IL17A, IL6, TNF	0.0002500
hsa04024	Signal transduction	cAMP signaling pathway	FOS, JUN, NFKBIA, RELA, CREB1, CALM1, MAPK1, NFKB1, PIK3CG	0.0012000
hsa04630	Signal transduction	JAK-STAT signaling pathway	JAK2, CSF2, EPO, IL10, IL6, PIK3CG, STAT3	0.0045000

## Data Availability

The data used to support the findings of this study are included within the article.

## Abbreviations

HH: Huangqi-Honghua herb pair
CI: Cerebral ischemia
TCM: Traditional Chinese medicine
ADME: Absorption, distribution, metabolism, and excretion
BMECs: Brain microvascular endothelial cells
OGD: Oxygen-glucose deprivation
H- C-T-D networks: Herb-compound-target-disease networks
KEGG: Kyoto Encyclopedia of Genes
GO: Genomes
PPI: Protein-protein interaction.
